# Editorial: Glutamatergic system in affective and psychotic disorders: pre-clinical and clinical advances

**DOI:** 10.3389/fnins.2023.1358271

**Published:** 2024-01-09

**Authors:** Dominik Strzelecki, Monika Talarowska, Jakub Kaźmierski, Napoleon Waszkiewicz, David Curtis

**Affiliations:** ^1^Department of Affective and Psychotic Disorders, Central Teaching Hospital, Medical University of Lodz, Łódź, Poland; ^2^Institute of Psychology, University of Lodz, Łódź, Poland; ^3^Department of Old Age Psychiatry and Psychotic Disorders, Central Teaching Hospital, Medical University of Lodz, Łódź, Poland; ^4^Department of Psychiatry, Medical University of Białystok, Białystok, Poland; ^5^UCL Genetics Institute, University College London, London, United Kingdom

**Keywords:** glutamatergic system, GABAergic system, schizophrenia, affective disorders, sarcosine, esketamine

The set of papers submitted for the Research Topic indicates the multifaceted nature of the involvement of the glutamatergic system in mental disorders and, more broadly, in the workings of the healthy brain, as well as various pathophysiological phenomena. It should come as no surprise, since the glutamatergic system is the largest neurotransmitter system of the human central nervous system, and glutamate, the neurotransmitter of this system, is the transmitter substance with the highest concentration in brain tissue. The glutamatergic system has an excitatory function, a kind of engine that, together with the GABAergic system, an excitatory regulator, and a brake, works out the balance and directs the information stream by harnessing other neurotransmitter systems to perform more specialized functions.

It is fair to say that for many decades, interest in the glutamate system has been inadequately low compared to neuroresearch concerning other systems with regard to its importance. It seems that the potential to exploit dopamine and serotonergic modulation in the current paradigm to date is depleting, hence the search for new therapeutic options has become a necessity. These studies have revealed a richer pathophysiological background for disorders such as depression and schizophrenia, as well as broadened the clinical applicability of psychiatric drugs.

In recent years, there has been particular interest in the inhaled form of esketamine in the treatment of drug-resistant forms of depression, glutamatergic modulators in the treatment of negative symptoms, and cognitive dysfunctions in schizophrenia and also dementia, such as Alzheimer's disease. While ketamine has been known in psychiatry for many years, simplifying the application procedure itself may help bring this drug into clinical practice. Esketamine has raised very high hopes for patients and doctors. Our experience shows the significant therapeutic potential of this substance. However, to evaluate the effectiveness of each new drug, we need at least a few years. The research presented in this Research Topic seeks to expand knowledge regarding its use in affective disorders. One paper in our issue compares the efficacy and safety of ketamine infusions in episodes of drug-resistant psychotic depression, as well as its effectiveness in recurrent depressive disorder and bipolar affective disorder (Gałuszko-Wȩgielnik et al.). The second paper (Marchi et al.) summarizes current knowledge on the effectiveness of ketamine and esketamine in improving anxiety, quality of life, and cognitive and social functioning.

We are at the beginning of the exploration of clinical interventions aimed at modulating the glutamatergic system. Currently available data allow us to make very general conclusions about the efficacy and safety of these therapies.

Even before the exploration of applications of glutamatergic modulation in affective disorders, there was much interest in the glutamatergic hypothesis of schizophrenia, related to NMDA (N-methyl-D-aspartate) receptor hypofunction. Research has shown that impairment of function of NMDA receptors located on GABAergic interneurons, rather than those on glutamatergic neurons, may be of special relevance, leading to inefficient inhibition of glutamatergic transmission and excessive information noise, among other things, in the hippocampus (Cohen et al., 2015; Strzelecki et al., 2015). The team coordinating this Research Topic is particularly interested in these topics, especially the involvement of the glutamatergic system in the pathogenesis of schizophrenia and the potential for NMDA receptor modulation in the treatment of negative symptoms and cognitive function, dimensions of the disease that largely determine the level of functioning and quality of life of patients with this diagnosis (Strzelecki et al., 2016; Curtis, 2019; Marchi et al., 2021; Rosenbrock et al., 2023).

Less often emphasized in etiopathogenetic considerations of schizophrenia, appearing to be very important, are changes involving dysfunction of the GABAergic system. Of particular importance is the decreased expression of glutamate decarboxylase—GAD67, the enzyme responsible for most of the production of gamma-butyric acid, in parvalbumin-positive cells (PV neurons), the same ones that are supposed to be the site of NMDA receptor hypofunction in schizophrenia (Curley et al., 2011; Dowling et al., 2023). Thus, we are dealing with two parallel processes impairing inhibitory processes—NMDA receptor hypofunction and decrease of GAD67 activity in GABAergic interneurons. Therefore, the modulation of the function of PV neurons should be the next target of intensive psychopharmacological research.

Sleep, like the glutamatergic and GABAergic systems, is waiting for its proper place on the research agenda. Phenomena related to sleep and its disorders, especially in the context of the importance of sleep in maintaining human mental and physical wellbeing remain largely unclear. Works that extend the research of various neurotransmission systems and new research methods of physiological and pathophysiological phenomena seem particularly interesting (Raitiere; Kaczmarski et al.).

Interesting perspectives arise from the possibility of reducing excitotoxic phenomena evidently associated with inadequately controlled glutamate release. Memantine is registered to treat more severe forms of Alzheimer's disease; while taking advantage of its good tolerability, we might use its protective properties to reduce the severity of toxic phenomena of both acute (stroke, trauma) as well as chronic in people with increasing organic changes of various origins (vascular, primary degenerative, in affective disorders or the preprodromal and prodromal periods of schizophrenia to reduce synaptic pruning), analogous to the use of low doses of acetylsalicylic acid in the prevention of cardiovascular disease (Strzelecki et al., 2013; Czarnecka et al., 2021; Martínez-Coria et al., 2021).

A separate place requires mention of the neurobiological and structural coexistence of the glutamatergic system with the GABAergic system, glial cells of the glial, and extracellular space (quadripartite synapse). Knowledge of these issues is very sparse, but clarification of the processes involved can point to new directions for diagnostic and therapeutic methods development, and we badly need it.

## Author contributions

DS: Writing – original draft, Writing – review & editing. MT: Writing – review & editing. JK: Writing – review & editing. NW: Writing – review & editing. DC: Writing – review & editing.
